# Illusory agency attribution to others performing actions similar to one’s own

**DOI:** 10.1038/s41598-019-47197-2

**Published:** 2019-07-24

**Authors:** Osamu Nomura, Taiki Ogata, Yoshihiro Miyake

**Affiliations:** 1 Department of Computational Intelligence and Systems Science, Tokyo Institute of Technology, Yokohama, Kanagawa Japan; 2 Department of Computer Science, Tokyo Institute of Technology, Yokohama, Kanagawa Japan

**Keywords:** Perception, Consciousness

## Abstract

When people observe others performing actions similar to their own while dancing or playing musical instruments, they sometimes feel as if their actions were subsumed into others’ actions or others’ actions led their own actions. Many studies have been conducted to investigate agency attribution. However, these studies have mainly examined agency attribution in cases where people do not know the true agent. Few studies have focused on how people attribute agency to others despite knowing that they themselves are actual agents. This study investigates agency attribution to others performing actions similar to one’s own when one knows who the actual agent is. We evaluated agency attribution when participants manipulated a mouse to control a cursor while observing another person performing similar actions. Our findings demonstrated that participants could attribute agency to others despite knowing that they themselves were actual agents. We refer to this illusory sense as “illusory agency attribution to others.” We suggest that illusory agency attribution to others is determined by multiple factors including a bottom-up process with a subjective feeling of agency in addition to a top-down process with an interpretative judgement of agency.

## Introduction

In daily life, people often have opportunities to interact with others while observing others performing actions similar to their own. For instance, people dance in groups while observing others dancing in a similar manner or play musical instruments in an orchestra while observing other musicians performing. In such situations, they sometimes feel as if their actions were subsumed into other people’s actions or that other people’s actions led their own actions. Under these circumstances, people clearly know that they caused their own actions and that the actions of others were not responsible for theirs. Despite this prior knowledge, is it really possible for people to have such an illusory sense that the actions of others were responsible for their own actions when observing others acting similarly?

It is well known that the sense of agency is one feeling that arises while performing actions. The sense of agency is a feeling that an observed outcome was caused by one’s own actions^1–3^. The sense of agency is caused by the integration of varied information related to causal actions, including efference copies of the motor command^4^ and visual or auditory sensory feedback about observed outcomes^3^. Sensory feedback that does not match the information on one’s own causal actions can reduce the sense of agency^2,5–12^. For instance, a previous study reported that incongruent sensory feedback can diminish the sense of agency^2^. In that study, participants explicitly reported a sense of agency when they observed a change in an image triggered by their own actions. In each trial, a small square piece appeared at the bottom of the monitor screen and moved straight upward at a uniform speed. When the participants pressed the key, the piece on the monitor screen jumped 35 mm upward, with varied temporal delays. The participants were instructed to report whether they felt they had made the piece jump as intended. The results revealed that the sense of agency weakened as the time lag increased. Further, a previous study^13^ investigated the effect of delays in visuomotor perception on the sense of agency. Participants manipulated a mouse to control a cursor on the screen and judged whether the cursor’s movements had been delayed and whether they felt that they had controlled the cursor. The results revealed a significant main effect of delay on the perception of delay as well as on the sense of self-agency. Hence, the sense of agency can be reduced by temporal inconsistency between visual information and actions.

The classical work by Michotte revealed the perception of causality based on spatial and temporal cues in the well-known launching effect^14^. Many studies have also been conducted to investigate the perception of causality between actor and outcome as “agency attribution” or “agency judgement”^1,6,15–22^. Those studies showed that individuals who perform actions could attribute the agency on their own actions to others. For instance, in a previous study^1^, the authors investigated agency attribution for a sound caused by pressing a button. In the congruent tone condition, pressing the right and left buttons evoked a specific corresponding tone, as in the learning session. By contrast, in the incongruent tone condition, a tone that differed from the tone evoked in the learning session followed the button press. Moreover, the onset of the tone was manipulated and varied. Congruency and delay were manipulated to evoke uncertainty concerning self-agency. Participants judged whether the sounds were activated by their own button press or a button press of the experimenter, who was seated in front of a computer behind a folding screen. In reality, the experimenter did not perform any action that produced the sound. Rather, all tones were generated by the computer and presented with a specific delay after participants pressed the button. However, participants were not informed of this. The results showed that the participants could attribute the button press to the experimenter in the incongruent tone and temporal delay conditions. In another study^17^, participants manipulated a joystick freely and observed the movements of a virtual joystick displayed on a monitor screen. Angular biases (spatial condition) or temporal delays (temporal condition) were imposed on the participants’ manipulation of the virtual joystick. Participants were asked to judge whether they were viewing the virtual joystick responding to their own movements (answer: “self”), modifications of their own movements (answer: “bias”), or the actions of another agent (answer: “other”). In the experiment, all the movements of the virtual joystick were in fact caused by the participants’ actions; however, participants were not informed of this. The results showed that the participants answered “other” for the highest biases while answering “self” for the lowest angular biases and “bias” for intermediate biases in the spatial condition. In summary, these studies showed that participants could attribute agency to others when the presence of another possible agent was suggested and participants did not know the true agent.

In a previous study^23^, the authors proposed a multifactorial weighting model to explain the sense of agency. This model indicated that the sense of agency comprises multiple processes: the feeling of agency with a bottom-up process produced by perceptual representation with sensory feedback or proprioception and the judgement of agency with a top-down process produced by propositional representation with contextual cues and thoughts. Based on the model, it is suggested that the participants in the previous studies judged others to be more plausible agents when they did not know the identity of the true agent, and agency attribution to others was caused primarily by a top-down process^1,6,15–22^.

In contrast, individuals generally know that they themselves are the true agents and that outcomes are caused by their own actions. In such situations, they could not judge others to be more plausible agents. If this is the case, can they attribute causal agency to similar actions by others? In our study, we aim to investigate agency attribution in the condition where people clearly know that they themselves are the true agents but are in the presence of others performing similar actions. Specifically, our goals are as follows: First, we address the question of whether people attribute agency to themselves based on their prior knowledge as agents or can attribute their agency to others performing similar actions, as in the condition in previous studies where the true agent was unknown. Second, we investigate whether agency attribution to oneself and to others are mutually exclusive or can co-occur when it is possible to attribute agency to others. Our final goal is to examine whether agency attribution to oneself and to others while observing the actions of others is a simple combination of agency attribution to oneself while working alone and agency attribution to others while merely observing the actions of others.

**Figure 1:**
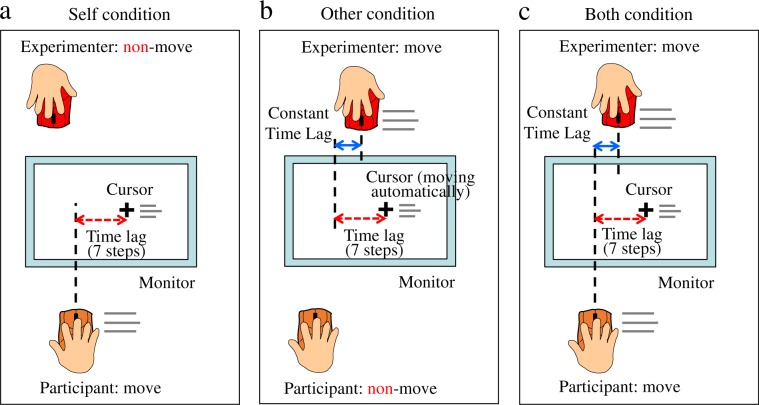
Experimental conditions. (**a**) In the Self condition, the participants moved the mouse to the right and left continuously with their dominant hand. The cursor controlled by the participants’ mouse moved on the horizontal center line on the monitor screen with preset time lags associated with the participants’ mouse. The experimenter held another mouse but did not move it. (**b**) In the Other condition, the participants held the mouse but did not move it. However, the cursor moved automatically based on data on participants’ mouse movements recorded during the practice period. The experimenter moved the mouse to the left and right continuously with his dominant hand just as the participants did in the Self condition. (**c**) In the Both condition, the participants performed the same actions as in the Self condition. The experimenter performed the same actions as in the Other condition. Thus, the Both condition combined the participants’ actions from the Self condition and the experimenter’s actions in the Other condition.

The experiment was conducted to study agency attribution under the following three conditions, which are shown in Fig. 1. During the task in all three conditions, the participants simultaneously observed the movements of the cursor on the monitor screen and the experimenter’s mouse. In the Self condition, the participants moved the mouse right and left continuously. The cursor controlled by the participants’ mouse moved along a horizontal center line on the monitor screen following a time lag associated with the mouse. The experimenter held another mouse but did not move it. In the Other condition, the participants held the mouse but did not move it. The cursor moved automatically based on data previously recorded during the practice period for participants to learn how to maniuplate the mouse. The experimenter moved his mouse left and right continuously, just as the participants did in the Self condition. In the Both condition, the participants performed the same actions as in the Self condition, while the experimenter performed the same actions as in the Other condition. We imposed time lags at 187-ms intervals (94, 281, 468, 655, 842, 1029, and 1216 [ms]) between the movements of the participants’ mouse and the cursor. We fixed a time lag of 377 ms between the movements of the participants’ mouse and the experimenter’s mouse in the Both condition. In the Other condition, we also fixed a time lag of 377 ms between the prerecorded movements of the participants’ mouse and the movements of the experimenter’s mouse. To evaluate participants’ agency attribution for each trial, we asked them to make a forced choice from the four answers “Myself,” “Experimenter,” “Both of us” and “Nobody” to the question “Who did you feel was controlling the cursor?” Our first and second goals were investigated by evaluating participants’ agency attribution in the Both condition. For the first goal, we predicted that participants would select the answer “Experimenter” and attribute agency to others performing actions similar to their own. For the second goal, we predicted that participants would select the answer “Both of us” and that agency attribution to oneself and to others would co-occur. Our final goal was investigated by comparing participants’ agency attribution to oneself in the Both and Self conditions and by comparing participants’ agency attribution to others in the Both and Other conditions. For the final goal, we predicted that agency attribution to oneself and to others in the Both condition would not be a simple combination of agency attribution to oneself in the Self condition and agency attribution to others in the Other condition.

## Results

In total, 24 individuals participated in the experiment. The data from four participants were excluded from the analysis. Three of these participants reported that they had lost their concentration during the experimental tasks. One participant selected the same answer in all trials in each condition (“Myself” in the Self and Both conditions and “Nobody” in the Other condition). The point of subjective equality (PSE)^10^, which is a criterion of subjective feeling, could not be calculated when all answers were identical. Thus, data from 20 participants were retained for analysis.

**Figure 2:**
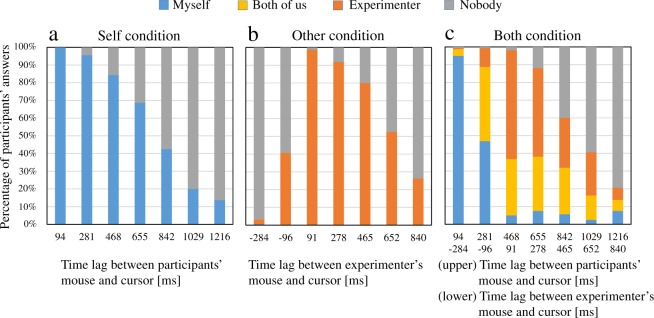
Percentage graphs of participants’ answers. (**a**) Self condition. (**b**) Other condition. (**c**) Both condition. The horizontal axis for the Self condition shows the time lags between participants’ mouse and the cursor, and the horizontal axis for the Other condition shows the time lags between the experimenter’s mouse and the cursor. The horizontal axis for the Both condition shows both of the time lag sets.

First, Fig. 2 shows the proportions of participant responses for each time lag in all three conditions. (see Supplementary Tables S1, S2 and S3). Figure 2 shows that the proportion of “Nobody” answers increased as the time lag increased in all conditions, except for negative lags between the experimenter’s mouse and the cursor in the Other condition. In addition, the answer “Experimenter” in the Both and Other conditions showed that the participants felt that the experimenter was controlling the cursor in some time lags, even though they knew this was not the case. Furthermore, in the Both condition, the participants selected the answer “Both of us.” This indicated that the participants felt that both they and the experimenter were jointly controlling the cursor. The proportion of “Both of us” answers in the Both condition decreased as the lag increased, except for negative lags between the experimenter’s mouse and the cursor.

**Figure 3:**
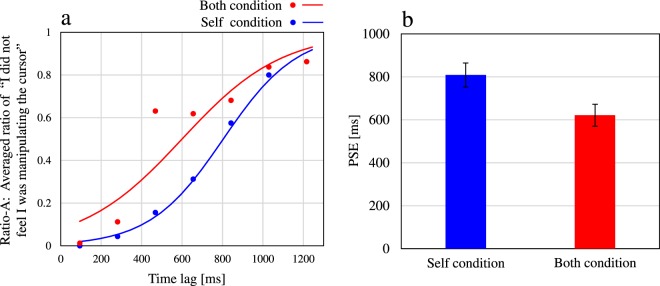
Evaluation of agency attribution to oneself. (**a**) Ratio-A: Average ratios of participants who did not feel that they were controlling the cursor for each time lag in the Both and Self conditions, including the answers “Experimenter” and “Nobody”. (**b**) The average PSEs in the Both and Self conditions. Error bars represent standard errors.

Next, we calculated Ratio-A in the Both and Self conditions. Ratio-A indicates the ratio of participants who did not feel they were controlling the cursor. This ratio was calculated as the sum of the proportions of answers “Experimenter” and “Nobody” in the Both and Self conditions, which excluded participants who felt that they were controlling the cursor. Figure 3a shows the average Ratio-A and the corresponding fitted logistic function curve. Along the vertical axis in Fig. 3a, lower values indicate that the participants felt they were controlling the cursor, while higher values indicate that the participants did not feel they were controlling the cursor. From Fig. 3a, we can deduce that participants lost the feeling of controlling the cursor in some time lags, even though they knew they were in fact controlling it. Thereafter, we estimated the time lag in which the ratio was 50% on the logistic function curve as the PSE to examine the differences between the curve shapes^10^. Figure 3b shows the average PSEs and the standard errors in the Self and Both conditions. A two-tailed t-test for pairwise comparisons between the Both and Self conditions was performed. Consequently, the PSE for the Both condition (mean = 621 ms, *SE* = 51 ms) was significantly smaller [*t*(19) = 4.82, *p* < 0.001, *d* = 0.78] than that for the Self condition (mean = 809 ms, *SE* = 56 ms).

**Figure 4:**
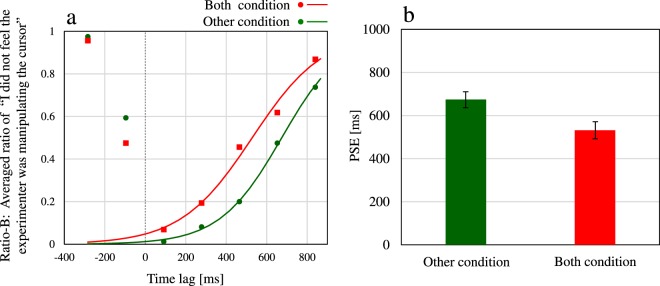
Evaluation of agency attribution to others. (**a**) Ratio-B: Average ratios of participants who did not feel the experimenter was controlling the cursor for each time lag in the Both and Other conditions, including the answers “Myself” and “Nobody”. (**b**) The average PSEs in the Both and Other conditions. Error bars represent standard errors.

Third, we calculated Ratio-B in the Both and Other conditions. Ratio-B indicates the ratio of participants who did not feel the experimenter was controlling the cursor. This ratio was calculated as the sum of the proportions of answers “Myself” and “Nobody” in the Both and Other conditions, which excluded participants who felt that the experimenter was controlling the cursor. Figure 4a shows the average Ratio-B and the corresponding fitted logistic function curve. In Fig. 4a, the horizontal axis represents the time lag between the cursor and the experimenter’s mouse. We fit the logistic function to only the ratios for the positive time lags between the cursor and the experimenter’s mouse. We discuss the ratios for the negative time lags in the discussion section. Along the vertical axis in Fig. 4a, a lower value indicates that the participants felt the experimenter was controlling the cursor, while a higher value indicates that the participants did not feel the experimenter was controlling the cursor. From Fig. 4a, we can deduce that participants had the feeling that the experimenter was controlling the cursor in some time lags, even though they knew the experimenter was not in fact controlling it. Thereafter, we estimated the PSE to examine the differences between the curve shapes. Figure 4b shows the average PSEs and the standard errors in the Both and Other conditions. A two-tailed t-test for pairwise comparisons between the Both and Other conditions was performed. Consequently, the PSE for the Both condition (mean = 531 ms, *SE* = 41 ms) was significantly smaller [*t*(19) = 3.46, *p* = 0.003, *d* = 0.83] than that for the Other condition (mean = 675 ms, *SE* = 37 ms).

Finally, we analyzed the actual time lag between the movements of the experimenter’s mouse and the participants’ mouse, which was expected to have a fixed value, in the Both and Other conditions. The average time lags between the movements of the experimenter’s mouse and the participants’ mouse in the Both and Other conditions were 419 ms (*SE* = 4 ms) and 425 ms (*SE* = 4 ms), respectively. A two-tailed t-test for pairwise comparisons with the Both and Other conditions as a factor was not significant [*t*(19) = 1.39; *p* = 0.180, *d* = 0.33]. No difference was found in the actual time lags between the Both and Other conditions. In addition, we analyzed the cycle of movements of the participants’ mouse in the three conditions, while only the cycle of movements in the Other condition were recorded during the practice period. The average cycle of the movements of the participants’ mouse in the Both, Self, and Other conditions were 1,415 ms (*SE* = 30 ms), 1,459 ms (*SE* = 63 ms), and 1,409 ms (*SE* = 43 ms), respectively. A one-way repeated-measures ANOVA with the condition as a factor was not significant [*F*(2,38) = 0.60, *p* = 0.497, *η*^2^ = 0.03]. No difference was found in the cycles of the movements of the participants’ mouse among the three conditions.

## Discussion

In this study, we aimed to investigate agency attribution to the similar actions of others in a condition where people clearly knew that they themselves were the true agents. Our first goal was to investigate whether people could attribute their agency to others performing similar actions to their own, even though they knew this was not the case. As shown in Fig. 4a, average ratios including the answers “Myself” and “Nobody”, where participants did not feel the experimenter was controlling the cursor in the Both condition, had small values on the small time lags. This result indicates participants felt that the experimenter was controlling the cursor even though they knew that he was not controlling it, unlike in previous studies^1,6,15–22^. Our second goal was to investigate whether agency attribution to oneself and to others are mutually exclusive or could co-occur in cases when it is possible to attribute agency to others. As shown in Fig. 2c, the answer “Both of us” was selected by participants in the Both condition. This result indicates that agency attribution to oneself and to others can co-occur. The feeling that the experimenter was controlling the cursor was contrary to fact. Therefore, we refer to it as “illusory agency attribution to others” in contrast to agency attribution to others in the previous studies. It is suggested that the feeling that one’s own actions were subsumed into those of others or that the actions of others led to one’s own actions while dancing or playing musical instruments in groups is caused by the co-occurrence of agency attribution to oneself and illusory agency attribution to others. This indicates that the boundary between the self and others could be ambiguous for agency attribution.

It was predicted in the model^23^ that no external attribution was caused as the feeling of agency with the bottom-up process based on mainly sensorimotor processes, and the judgement of agency was formed as the interpretative judgement of being the agent with the top-down process based on mainly conceptual and meta-representational processes. The illusory agency attribution to others observed in our study can also be considered a top-down process because the results reflected less attribution on the negative time lags between the cursor and the experimenter’s mouse than on the positive time lags in the Other and Both conditions, as shown in Fig. 4a. The negative time lags between the cursor and the experimenter’s mouse indicate that the cursor movements preceded the experimenter’s mouse movements. However, it was usually impossible for the cursor movements to precede the mouse movements. Thus, we assume that illusory agency attribution to others was less pronounced for negative time lags because of this prior knowledge. Illusory agency attribution to others, however, was evaluated as a subjective feeling, not just as a judgement, since we asked for participants’ feelings about the control of the cursor in our experiment. In addition, the participants clearly knew that the experimenter was not the true agent, and thus, they did not judge others to be more plausible agents for the attribution, unlike in previous studies^1,6,15–22^. Specifically, the illusory agency attribution to others was a subjective feeling that involved the feeling of agency with the bottom-up process in the aforementioned model. As a result, we can conclude that the illusory agency attribution to others can be determined by multiple factors, including bottom-up and top-down processes. The illusory agency attribution to others observed in our study could be different from agency attribution to others, which was mainly based on the top-down process, observed in previous studies^1,6,15–22^.

Our final goal was to investigate whether agency attribution to oneself and to others while acting by oneself and observing the actions of others is a simple combination of agency attribution to oneself while working alone and agency attribution to others while merely observing the actions of others. We found that the PSE of agency attribution to oneself in the Both condition, in which another person was performing actions similar to those of the participant, was significantly smaller than the PSE in the Self condition, in which no other person’s actions took place. This smaller PSE indicates that the participants lost their feeling that they were controlling the cursor in the case of smaller time lags in the Both condition. In addition, we found that the PSE of illusory agency attribution to others in the Both condition was significantly smaller than the PSE in the Other condition, in which participants had no control over the cursor. This smaller PSE indicates that the participants lost their feeling that the experimenter was controlling the cursor in the case of smaller time lags in the Both condition. Based on these results, we discuss a process for agency attribution to oneself and illusory agency attribution to others. The input compared with the outcome (cursor movements) for agency attribution to oneself was the information related to participants’ actions, including efference copies of the motor command and proprioception. In contrast, the input compared with the outcome for illusory agency attribution to others was the visual information related to the experimenter’s actions. Thus, the different inputs were compared with the outcome for each type of agency attribution. Assuming that brain processes responsible for each type of agency attribution differed due to those different inputs, each type of agency attribution would arise independently. In this case, a simple combination of different attributions would be observed in the Both condition, where the different inputs co-occurred; the PSE of the agency attribution to oneself would not have differed between the Both and Self conditions, and the PSE of the illusory agency attribution to others would not have differed between the Both and Other conditions. Contrary to these predictions, the differences were found between the respective PSEs in our results. As shown in Fig. 2, the reason why the PSEs of agency attribution to oneself differed is mainly that “Experimenter” answers increased in the Both condition. Furthermore, the reason why the PSEs of the illusory agency attribution to others differed is mainly that “Nobody” answers increased more in the Both condition than in the Other condition. Those differences in the PSEs suggest that each form of agency attribution did not arise independently in the Both condition. Rather, the illusory agency attribution to others in the condition when participants and others performed similar actions differed from agency attribution while just observing another person’s actions. In addition, the agency attribution to oneself in the condition when participants and experimenters performed similar actions differed from agency attribution while acting alone without observing the actions of others. Consequently, we found that each agency attribution observed in the Both condition in our study could be specific to the condition where similar actions of oneself and others co-exist. In other words, agency attribution to oneself and to others in the Both condition would not be a simple combination of agency attribution to oneself in the Self condition and agency attribution to others in the Other condition.

Many previous studies related to the observation of others’ actions have been reported based on the mirror neuron system view. These studies showed that the premotor cortex and inferior parietal lobule are involved in the mirror neuron system^24–30^. It is known that these parts of the brain are activated both when people are only observing similar actions of others and when they themselves are acting. At the same time, the premotor cortex is a part of brain related to action planning and is activated when people feel the sense of agency^16,31^. Moreover, the inferior parietal lobule is known to play an important role in the sense of agency to evaluate the temporal consistency of sensory information^3,16,31,32^. Thus, the premotor cortex and inferior parietal lobule can be activated by one’s own actions and similar actions by another person. Specifically, previous studies reported that the inferior parietal lobule was activated when agency was externally attributed^3,16,33,34^. This indicates that the activated inferior parietal lobule in the mirror neuron system could also contribute to agency attribution to others. The subjective feelings underlying illusory agency attribution to others can be explained as characteristics of the mirror neuron system, which can cause vicarious feelings, as reported in previous studies^35–40^. These findings suggest that some interactions take place between the processes of agency attribution to oneself and illusory agency attribution to others when people are acting while observing similar actions performed by others. These interactions can be one of the reasons for the difference in agency attribution to oneself between the Both and Self conditions and the difference in illusory agency attribution to others between the Both and Other conditions.

Additional questions concerning illusory agency attribution to others could be raised. Did illusory agency attribution to others arise when participants lost agency attribution to themselves in the Both condition? This was not the case because participants selected the answer “Nobody” in certain cases, indicating that participants lost the agency attribution to themselves and also the illusory agency attribution to others. In these cases, to whom was their agency then attributed? There were two possibilities: invisible others and the cursor moving automatically. After the experiment, some participants reported that they felt the cursor was moving automatically, even though they knew that they themselves were controlling the cursor in the Both condition. Therefore, we speculated that participants felt the cursor was moving automatically when they answered “Nobody”, unlike previous studies in which agency was attributed to invisible others^1,17^. We could investigate the loss of agency attribution in detail by using the answers “Invisible other” and “Cursor moving automatically” instead of the answer “Nobody.”

An argument could be raised that the illusory agency attribution to others was merely the result of demand characteristics or response biases, as the participants answered the explicit question concerning agency attribution with knowledge of the actual agent. In a previous study of the sense of agency^41^, participants’ explicit self-reports of the sense of agency were evaluated for the same condition as in our study, i.e., the condition in which the participants knew the actual agent (themselves). The study also evaluated intentional binding, which was considered an implicit measure of the sense of agency^42^ and showed overall that the explicit self-reports of the sense of agency correlated with intentional binding on consistency between action and outcome, with some differences. Thus, participants’ explicit self-reports with the knowledge of an actual agent can indeed measure a sense of agency and do not necessarily induce demand characteristics or response biases.

One participant excluded from the analysis selected the same answer in all trials in each condition. His/her answers would be strongly affected by the initial instructions that he/she was the agent in the Self and Both conditions and that the cursor moved automatically in the Other condition. This result suggests that some people’s responses would be influenced strongly by a top-down process with prior knowledge. In a future work, objective measures such as intentional binding should be used to investigate the illusory agency attribution of these people.

The introduction of implicit or objective indicators may help investigate illusory agency attribution to others under more natural conditions including sports or musical performances. In our study, the participants could select the answer “Both of us.” We speculate that this answer could be related to the feeling of unity with other people. This feeling could be helpful for group dances or musical performance in an orchestra. However, this feeling could adversely affect performance in individual competitions. Future work should investigate how illusory agency attribution to others affects performance in music and sports.

In this study, we investigated agency attribution to others in a condition in which individuals clearly knew that they themselves were the true agents. In conclusion, first, we found that participants attributed agency to others performing similar actions to their own despite knowing that they themselves were the actual agents. We referred to this illusory sense as illusory agency attribution to others. Second, we found that illusory agency attribution to others could co-occur with agency attribution to oneself. Finally, we found that agency attribution while acting by oneself and observing the actions of others is not a simple combination of agency attribution while working alone and while merely observing the actions of others.

## Methods

### Participants

A total of 24 people (3 women and 21 men, mean age: 25 years, age range: 21–59 years) participated in the experiment. They were all right-handed and had normal hearing and normal or corrected-to-normal vision. They were not informed of the purpose of the experiment. Written informed consent was obtained, and the participants were compensated. The sample size for the two-tailed t-test for pairwise comparison of PSEs was estimated to be nineteen by an a priori power analysis (*d* = 0.7, *α* = 0.05, *Power* = 0.8). Therefore, we considered that the data from twenty participants after excluding the data from four participants was sufficient. This experiment was approved by the Tokyo Institute of Technology’s Ethical Review Board for Epidemiological Studies and undertaken in accordance with the approved guidelines.

### Apparatus and Stimuli

**Figure 5:**
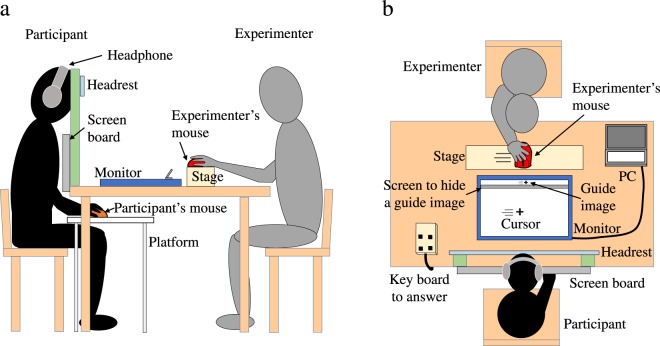
Experimental system. (**a**) Side view. A platform for the mouse was set under the desk on the participants’ side. The participants’ hand and the mouse were thus hidden from the participants’ line of sight. During the task, the participants observed the movement of the cursor on the monitor screen and the movement of the experimenter’s mouse simultaneously. (**b**) Top view. On the experimenter’s side, a stage to move the mouse was set along the length of monitor screen. On the screen on the experimenter’s side, a small guide image, hidden from participants’ sight by a low screen, was displayed for the experimenter to follow.

Participants used a USB computer mouse (GM299, TeckNet, UK). The experimenter used a different USB computer mouse (MA-BL9R, Sanwa, JP), which never affected the cursor movements. A cursor image was shown on an LCD monitor (Diamondcrysta RDT233WLM, Mitsubishi, JP), with a resolution of 1024 × 768 pixels and refresh rate of 60 Hz (see Fig. 5). A metronome beat (MA-1, KORG, JP) of 45 BPM was delivered during the practice period to train the participants to move the mouse at regular intervals.

**Figure 6:**
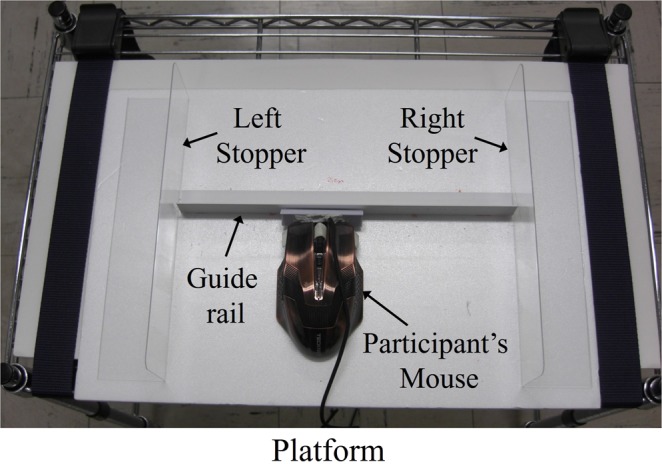
Platform for participants to move their mouse. The guide rail and the right and left stoppers on the platform were set to guide the participants to move the mouse parallel to the length of the monitor screen.

The movement of the cursor was delayed by a time lag following the movement of the mouse, which was controlled by a purpose-driven program we developed in Visual Basic 2013 on a PC (Surface Pro 2, Microsoft, USA). This program allowed cursor movements to include an 8-ms delay to mouse movements due to a data transmission lag with a report rate of 125 Hz. The cursor was cross-shaped and was 7 mm × 7 mm in size. It was black to contrast with the white background of the screen. The guide image for the experimenter to follow with his mouse was displayed on the experimenter’s side of the monitor screen (see Fig. 5b) and had the same shape and the colour as the cursor. The length between the right and left stoppers limiting where the mouse could move on the platform was 30 cm (see Fig. 6), and the length of the available area for cursor movement on the monitor screen was also 30 cm.

### Task and Condition

The participants moved the mouse until they touched the stoppers on the right and left sides of the platform in the Self and Both conditions. They repeated this action five times until a beep sounded. The experimenter moved his mouse on the stage until the mouse reached the marker lines on both ends in the Other and Both conditions. The time lags between the movements of the participants’ mouse and the cursor were set at 187-ms intervals (94, 281, 468, 655, 842, 1029 and 1216 [ms]). The time lags between the expected movements of the experimenter’s mouse and the cursor were set at 187-ms intervals (−284, −96, 91, 278, 465, 652, and 840 [ms]). The movements of the experimenter’s mouse followed the small guide image displayed on his side of the monitor screen to perform actions similar to the participants’ actions. We estimated the reaction time for an experimenter to recognize the onset of the guide image as approximately 200 ms^43,44^. We intended to set the time lags between the expected movements of the experimenter’s mouse and the cursor to be close to the time lags between the movements of the participants’ mouse and the cursor in part. In this case, the fixed time lag between the movements of the experimenter’s mouse and the participants’ mouse should equal approximately an integral multiple of the time interval (187 ms). Hence, we set the fixed time lag to 377 ms, which was the closest value to twice the time interval, while considering a configurable minimum time step. The guide image was set to a 177 ms constant delay following the movement of the participant’s mouse for the Both condition and following the prerecorded movements of the participants’ mouse for the Other condition. Actual time lags between the movements of the experimenter’s mouse and the participants’ mouse were not indeed constant, but reflected the inevitable variance of the movements of the experimenter because the experimenter followed the guide image. The actual time lags were recorded and analysed following the experiment. Negative values for time lags indicated that the cursor movements preceded the experimenter’s mouse movements. From these time lag sets, we were able to evaluate 7 time lags between the participants’ mouse and the cursor as well as between the experimenter’s mouse and the cursor.

### Procedure

The experiments were conducted in a dark and soundproof room. The experimenter and participant faced each other across a desk (60 cm apart) on which an LCD monitor was placed (see Fig. 5a). On the participant’s side, the platform where the mouse would be used (59 cm in height) was set under the desk (see Fig. 5a); therefore, the participant could not see either the mouse or his or her own hand. A guide rail and right and left stoppers were set to help the participants move the cursor along the long axis of the monitor screen (see Fig. 6). On the experimenter’s side, a stage for moving the mouse was set along the length of the monitor screen (width: 9.0 cm, height: 5.7 cm). On the experimenter’s side of the monitor screen, a guide image hidden from the participants’ sight by a low screen, shown in Fig. 5b, was displayed for the experimenter. The experimenter performed actions similar to those of the participant by following this guide image with his own mouse. The participants were clearly informed that the movements of the cursor on the monitor screen were caused by their own mouse movements or by prerecorded data on participants’ movements during the practice period. Thus, the participants knew that the movements of the experimenter’s mouse never affected the cursor movements. On the participant’s side, a head rest and a chin rest were positioned to hold the participant’s head still. (The head rest is shown in Fig. 5a, but the chin rest is not shown in the figure to avoid complicating the drawing). During the experiment, each participant placed his or her chin and forehead on the chin rest and head rest, respectively. Using the chin rest and head rest, the distance between the center of the screen and the center of the participant’s eyes was fixed at about 42 cm, and the angle of the participant’s view to the horizontal axis was fixed at about 50 degrees. A screen board was set under the desk to hide the movements of the participant’s arm from his or her own line of vision. Therefore, the participants were not able to see their own hand or arm movements during the task. They were also asked to put on headphones with white noise playing to mask the sound generated by the movement of the mouse on the platform.

First, the participants underwent one set of trials with the seven time lags for each condition as practice. During this practice period, the participants learned to move the mouse from left to right and from right to left following the beat of a metronome. The order of three conditions was fixed as follows in the practice period:

Self condition → Other condition → Both condition

For the Self condition in the practice period, the movement data from the participant’s mouse were recorded on a PC and later used to move the cursor automatically in the Other condition. We determined the values of the time lag and the pitch of continuous mouse movements to avoid movements with half-cycle differences between the participants’ mouse and the cursor because such reversals could also affect the sense of agency^45^. Then, the main experimental rounds were conducted after the practice period. The order of the three conditions was counterbalanced among the participants. Intersession breaks of about 10 minutes were given between each condition. The participants performed eight sets of trials consecutively for each condition. One set of trials consisted of the seven time-lag values. The order of the time lags in the trials was randomized. In total, each participant performed 56 trials for each condition (168 trials in total).

**Figure 7:**
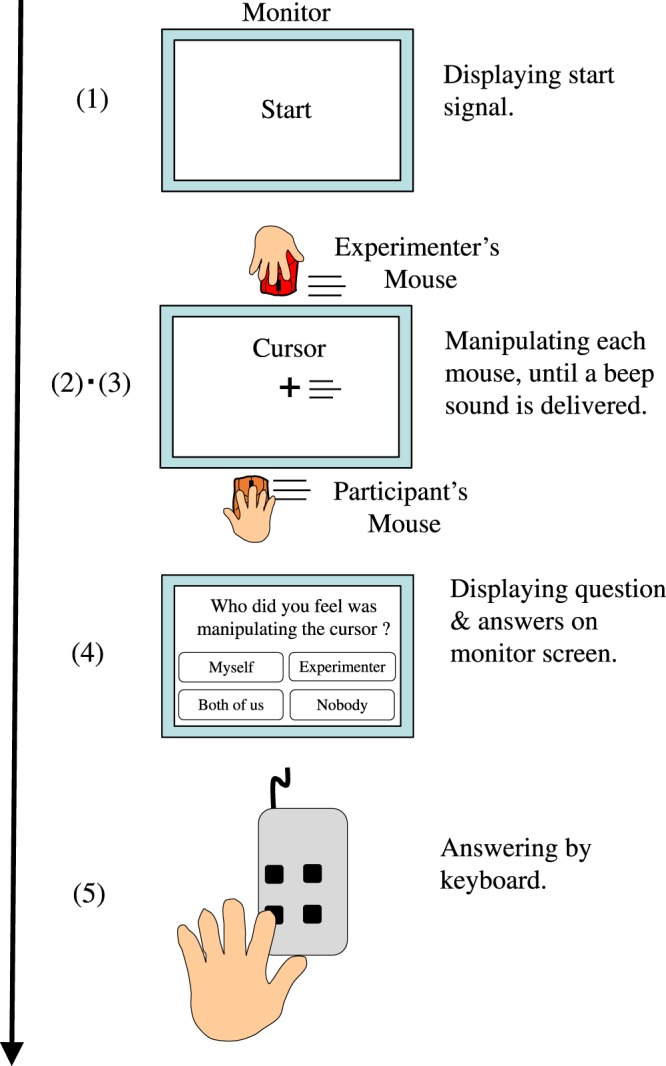
Order of the procedures for each trial for each time lag. (1) The word “Start” was displayed on the monitor screen to instruct the participants to start moving their mouse. (2) The participants moved their mouse to control the cursor in the Both and Self conditions, and the cursor moved automatically in the Other condition. The experimenter moved his mouse using similar actions in the Both and Other conditions. (3) The participants stopped moving their mouse when a beep sounded after the mouse had been moved five times to the right and left in the Both and Self conditions, and the cursor stopped automatically after moving five times to the right and left in the Other condition. (4) The question and answers were displayed on the screen. (5) The participants selected the answer that best matched their feeling from the four options provided by pressing the key.

The participants were asked to make a forced choice from four possible answers to evaluate their agency attribution. The prompt and four answer options were displayed on the monitor screen after each trial. The answers were recorded by pressing a key. The four answers were displayed at the corner of the rectangle, and the participants pressed the direction key corresponding to the positions of the answers on the monitor screen. The positions of the answers and keys were changed after every predetermined number of trials. Each trial for each time lag was executed in the order shown in Fig. 7.

### Statistical analysis

We calculated the following two ratios, combining the answers to evaluate the attenuation of agency attribution to oneself (Ratio-A) and to others (Ratio-B).

- Ratio-A: The ratio of participants who did not feel they were controlling the cursor.
- Ratio-B: The ratio of participants who did not feel the experimenter was controlling the cursor.

We plotted these ratios and fitted them to a logistic function. Furthermore, we estimated the time lag where the ratio was 50% on the logistic function curve as the PSE to examine the differences between the curve shapes using the following formula^10,46^.

1 $$R(t)=\frac{1}{1+\exp (\,-\,a(t-{t}_{{\rm{PSE}}}))}$$

where *t* is the time lag, and *R(t)* is the ratio, *a* indicates the steepness of the fitted curve, and *t*_PSE_ indicates PSE, representing the time lag where the ratio is 50%. In our experiment, *t* served as an independent variable, and *R(t)* was the observed data. The fitting was performed using a nonlinear least squares method.

The PSEs were tested for normality using the Kolmogorov-Smirnov test and then statistically analyzed with a two-tailed t-test for pairwise comparisons. The average time lags between the movements of the experimenter’s mouse and the participants’ mouse were calculated for each participant in the Both and Other conditions. The average time lags were tested for normality using the Kolmogorov-Smirnov test and then statistically analyzed with a two-tailed t-test for pairwise comparisons. The average cycles of the movements of the participants’ mouse were calculated for each participant. To evaluate sphericity, the Greenhouse-Geisser method was used. The average cycles were statistically analyzed with a one-way repeated-measures ANOVA with the condition as a factor. The significance level *α* was set at 0.05 for all tests. The effect sizes for the t-test were calculated as Cohen’s *d*, and the effect size for the ANOVA was calculated as a squared correlation ratio. All statistical tests were performed using the MATLAB Statistics and Machine Learning Toolbox (MathWorks, Natick, MA, USA), and an a priori power analysis was performed using G*Power^47^.

## Supplementary information

Supplementary information

## Data Availability

The datasets generated during and/or analyzed during the current study are available from the corresponding author on reasonable request.
