# Supplementary material for: Illusory agency attribution to others performing actions similar to one’s own
Source: Sci Rep. 2019 Jul 24;9:10754. doi: 10.1038/s41598-019-47197-2 (PMC6656881; doi:10.1038/s41598-019-47197-2)
Supplement: Supplementary file 1 — Supplementary information [file 41598_2019_47197_MOESM1_ESM.pdf]

Illusory agency attribution to others performing actions similar to one's own

Osamu Nomura[1], Taiki Ogata[2], and Yoshihiro Miyake[2]

[1] Department of Computational Intelligence and Systems Science, Tokyo Institute of Technology, Yokohama, Kanagawa, Japan.

[2] Department of Computer Science, Tokyo Institute of Technology, Yokohama, Kanagawa, Japan.

Correspondence and requests for materials should be addressed to O.N. (email: nomura.osamu@myk.dis.titech.ac.jp)

Supplementary Table S1. Proportions of participant's answers in the Self condition.

Answer \ Time lag [ms]	94	281	468	655	842	1029	1216
Myself	1.00	0.96	0.84	0.69	0.43	0.20	0.14
Experimenter	0.00	0.00	0.00	0.00	0.00	0.00	0.00
Both of us	0.00	0.00	0.00	0.00	0.00	0.00	0.00
Nobody	0.00	0.04	0.16	0.31	0.57	0.80	0.86

Supplementary Table S2. Proportions of participant's answers in the Other condition.

Answer \ Time lag [ms]	-284	-96	91	278	465	652	840
Myself	0.01	0.00	0.00	0.00	0.00	0.00	0.00
Experimenter	0.03	0.41	0.99	0.92	0.80	0.53	0.26
Both of us	0.00	0.00	0.00	0.00	0.00	0.00	0.00
Nobody	0.96	0.59	0.01	0.08	0.20	0.47	0.74

Supplementary Table S3. Proportions of participant's answers in the Both condition.

Time lag [ms] / Answer	94 -284	281 -96	468 91	655 278	842 465	1029 652	1216 840
Myself	0.94	0.47	0.05	0.07	0.06	0.03	0.08
Experimenter	0.01	0.10	0.61	0.50	0.28	0.24	0.07
Both of us	0.04	0.42	0.32	0.31	0.26	0.14	0.06
Nobody	0.01	0.01	0.02	0.12	0.40	0.59	0.79
